# Totally endoscopic non-robotic excision of aortic valve fibroelastoma: a case report

**DOI:** 10.1186/s13019-022-02040-0

**Published:** 2022-11-19

**Authors:** Sadia Batool, Arnaud Patoir, Amelie de Meaux, Marco Vola

**Affiliations:** 1 Department of Cardiac Surgery and Lung and Heart Transplantation, Hospices Civils de Lyon, Hôpital Louis Pradel, 28 Avenue du Doyen Lépine, 69677 Bron Cedex, France; 2 Department of Thoracic Surgery and Lung Transplantation, Hospices Civils de Lyon, Hôpital Louis Pradel, 28 Avenue du Doyen Lépine, 69677 Bron Cedex, France; 3 Echocardiography Unit, Hospices Civils de Lyon, Hôpital Louis Pradel, 28 Avenue du Doyen Lépine, 69677 Bron Cedex, France

**Keywords:** Papillary fibroelastoma (PFE), Aortic valve (AV), TEAVR (totally endoscopic aortic valve replacement)

## Abstract

**Background:**

Papillary fibroelastomas (PFEs) are a rare subtype of benign primary cardiac tumours, which are most commonly found on the aortic valve. Although median sternotomy is still used frequently there has been different attempts to remove the aortic valve PFEs minimally invasively using robotic and Mini sternotomy approach.

**Case presentation:**

We report herein a case of totally endoscopic non robotic removal of PFE of aortic valve.

**Conclusions:**

The encouraging intra and post-operative outcomes and fast recovery using totally endoscopic approach for removal of PFE shows the potential benefits of this technique.

**Supplementary Information:**

The online version contains supplementary material available at 10.1186/s13019-022-02040-0.

## Background

Although the prevalence of primary cardiac tumours ranges from 0.0017 to 0.28%, the papillary fibroelastomas (PFE) are second most common benign neoplasm of the cardiac valves after myxomas [1]. Despite the benign nature of this tumour, it carries very high risk of embolic complications including neurological deficit. The fragile nature and frond-like papillary tissues of the tumour itself is prone to thromboembolism. Therefore, once diagnosed, urgent surgical management is indicated even in asymptomatic patients [1]. Usually, PFEs are excised via sternotomy. Minimally invasive cases have been reported, we present here a case of totally endoscopic resection of fibroelastoma which to our knowledge is the first non-robotic that is reported.

## Case presentation

An asymptomatic 70-year-old women with hypertension, diabetes mellitus and hypercholesterolemia was referred for an aortic valve mass detected by a routine echocardiography. Physical examination was normal and BMI (Body mass index) 26.3. Pre operative blood test showed haemoglobin of 122 g/l, leucocytes 6.9 giga/l, INR 2.4, platelets 256 giga/l. The infectious serology was negative. Patient was started on warfarin 5 mg.

**Fig. 1:**
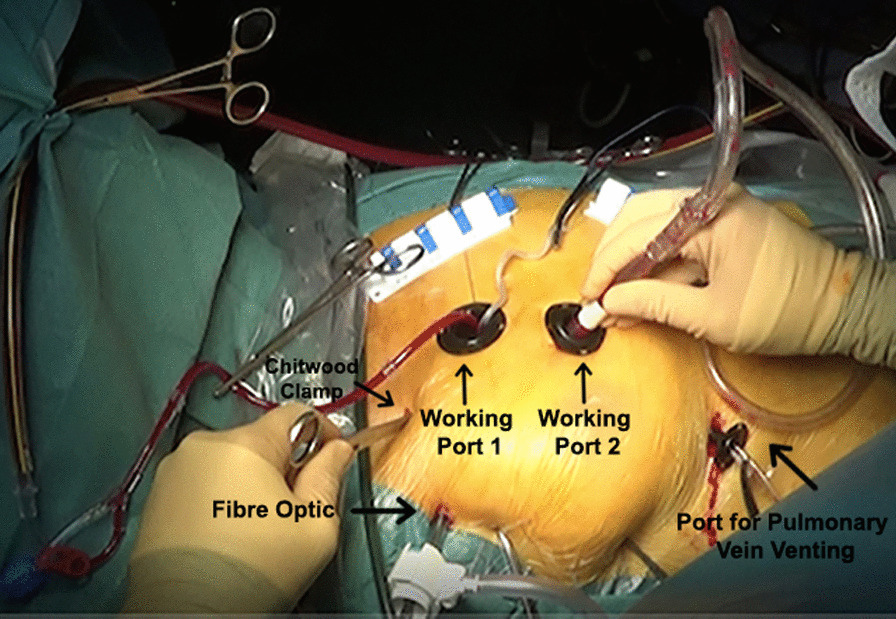
Operating field and trocar positioning

**Fig. 2:**
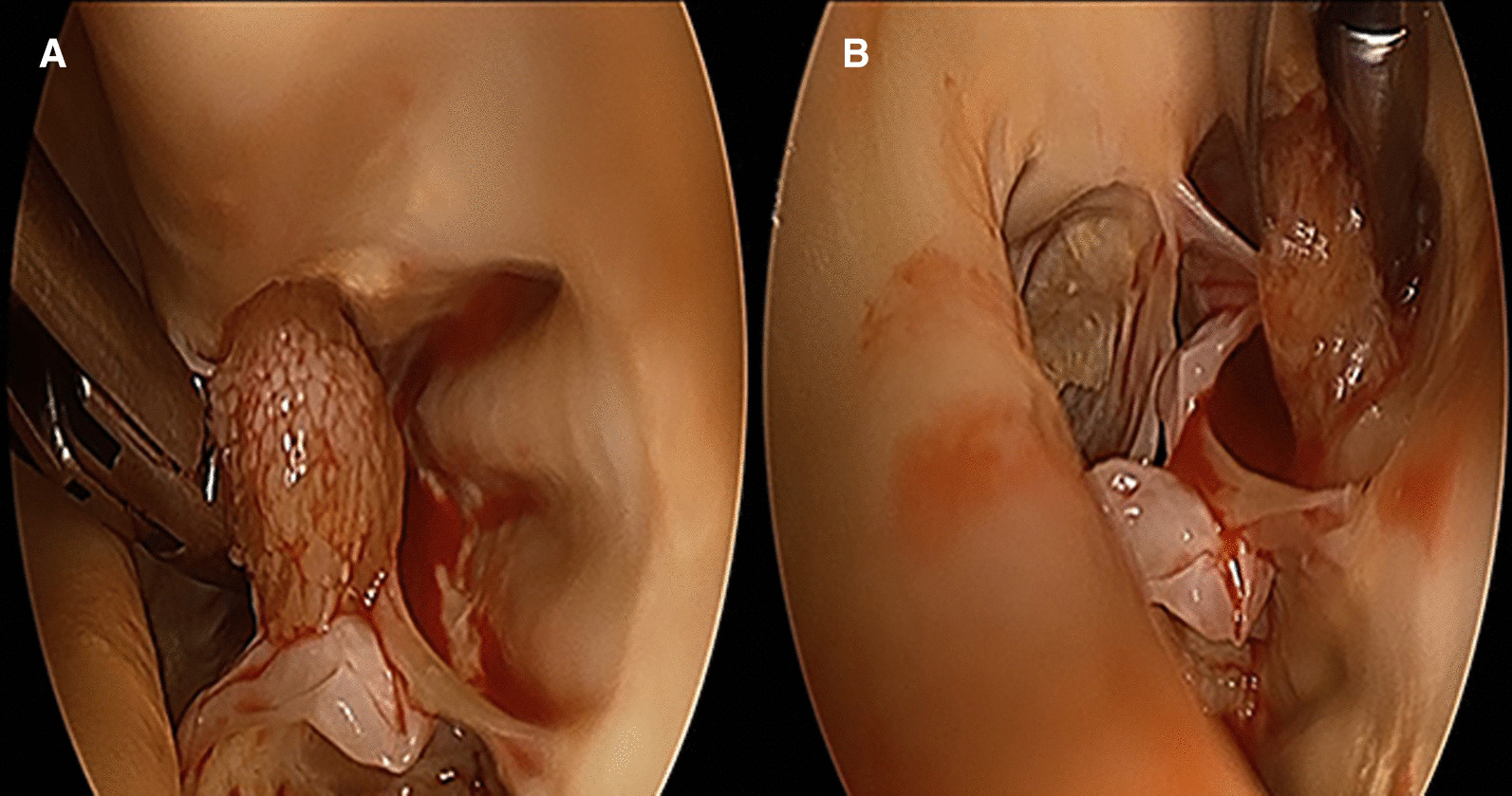
**A** Fibroelastoma on the right coronary cusp. **B** view showing the implantation of fibroelastoma on the right coronary cusp between the nodule of Arantius and the commissure

**Fig. 3:**
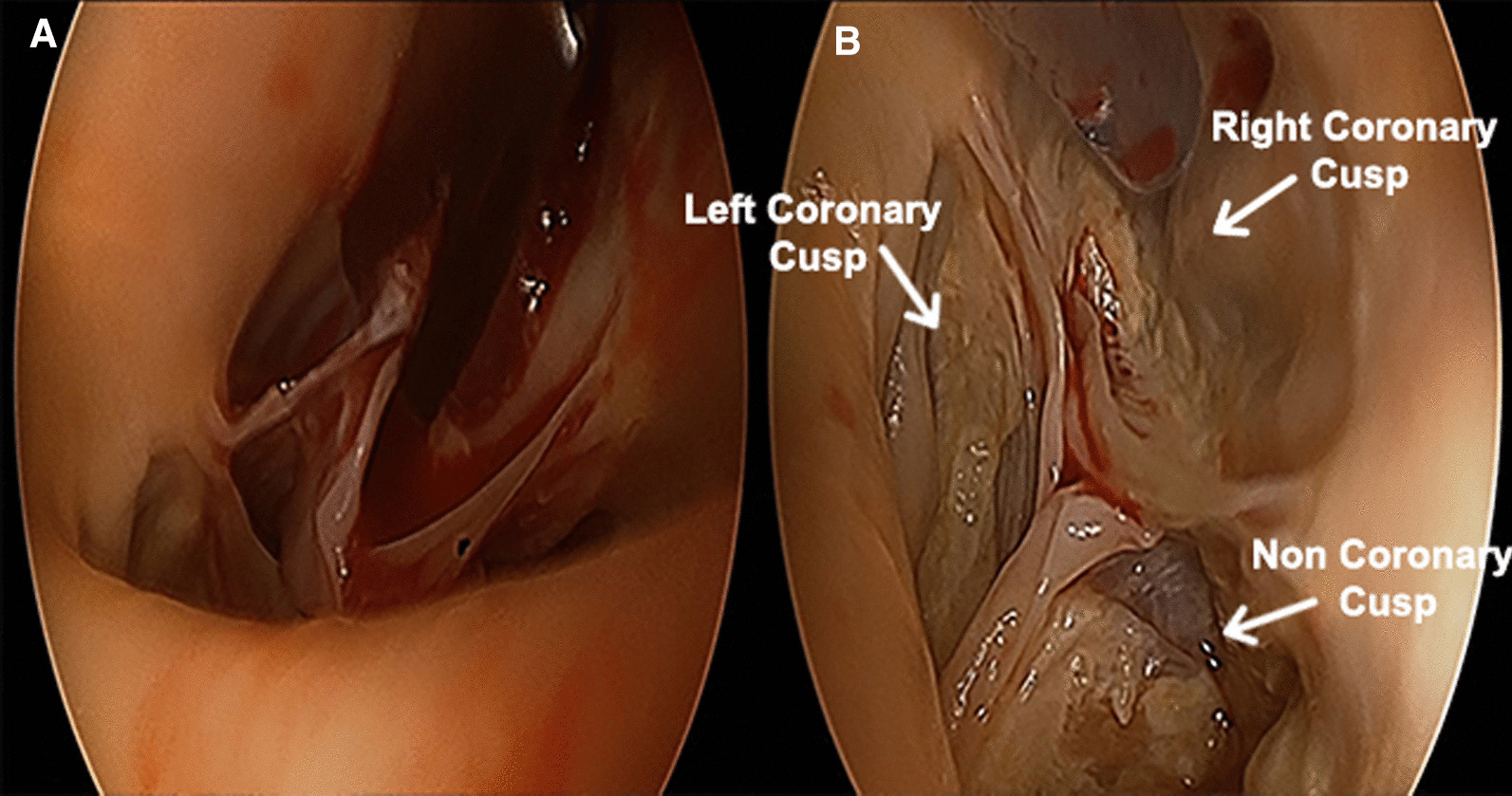
**A** Image showing a precise excision of the mass and its pedicle. **B** post-excision aortic valve view

Echo showed a mobile mass of 12 × 13 mm attached to the right coronary cusp diagnosed as a PFE. Aortic valve was tricuspid with mild aortic and mild mitral insufficiency pre op. Patient had ejection fraction of 75% and cardiac index of 2.2 l/min/m^2^. Given the risk of a potential embolization, the patient was referred for surgical resection. Pre-operative transoesophageal echocardiography confirmed the implantation of the mass to the right coronary cusp free edge. Surgical setting reproduced endoscopic triangulation as reported in totally endoscopic aortic valve replacement (TEAVR) (Fig. 1) [2]: a right anterior skin incision of 2 cm was done at second intercostal space (main working port), a second working port was located in the 3rd space. Five mm ports were added for a Chitwood clamp (1st intercostal space), camera (2nd) and left pulmonary vein vent (5th). Intervention was carried out under normothermia via a femorofemoral cardiopulmonary bypass (CPB). After traction under endoscopic detailed visualization of the free edge of the valve and of the implantation (Fig. 2) the mass and its pedicle were excised (Fig. 3a). Visual result and saline test, performed by filling the aortic root while venting the left ventricle, were judged satisfactory (Fig. 3b). The operative steps are shown in detail in the video (Additional file 1). Aortic clamp and CPB times were 48 and 79 min respectively. Mechanical ventilation time was 3 h and drainage 50 ml in 24 h. The patient was discharged at post-operative day 7. Post-operative trans-oesophageal echocardiography control showed mild to moderate aortic insufficiency which remained stable at 1-year follow-up.

The histopathology report confirmed the mass as fibroelastoma measuring 1 cm of maximal transverse diameter. At 1 year of follow-up the patient is asymptomatic with an active lifestyle. Aortic regurgitation and left ventricle dimensions and function are unvaried as compared to preoperative measures, with no signs of tumoral re-growth.

## Discussion and conclusion

Papillary fibroelastomas (PFE) are rare primary tumours of cardiac origin accounting for approximately 10% of all primary cardiac neoplasms [3]. They may occur anywhere on the endocardium, although are more common in the left heart and display a predilection for valvular structures. The aortic valve is the most commonly affected, with a number of such tumours arising from the non-coronary leaflet. Whilst the majority are incidental findings on echocardiography, symptoms most commonly occur subsequent to embolization, and may give rise to a wide variety of presenting features including neurological events (transient ischaemic attack, stroke, amaurosis fugax, spinal cord infarction), acute coronary syndrome, and distal thromboembolism [3]. PFES removal may require, when the implantation on the valvular tissue is large, extensive aortic valve reconstruction or aortic valve replacement.

In our patient PFE base was implanted exclusively on the free edge of the right coronary cusp with a fine pedicle and a low probability of needing a complex aortic valve reconstruction after resection. Some anatomical criteria, like non-obesity BMI, and the aorto iliac suitability for a retrograde arterial perfusion, encouraged us to consider an endoscopic minimal access targeting a superior post-operative early life quality rather than a ministernotomy.

Although median sternotomy is the usual approach, removal of the aortic valve PFEs by ministernotomy or robotic approach have been reported [3, 4].

Robotic adoption in totally endoscopic aortic valve setting has been shown to be very promising when performed in specialized centres [4]. In theory, robotic setting, considered the high grade of liberty of the tips of the instruments, should provide the possibility of more technically demanding aortic valve free margin reconstruction, or larger indications in more challenging anatomical settings (e.g., obesity). But reports are too rare to any statistical conclusion, and the cost of the robotic equipment may limit the adoption in small centres.

Totally endoscopic cardiac surgery has shown to improve early life quality when compared with open full sternotomy and minithoracotomy approaches for myocardial revascularization and atrial septal defect repair and it also showed successful aortic valve replacement in a cohort of selected low risk patients with very encouraging results in terms of post-operative paravalvular leakages, conduction blocks and early life quality [2].

Based on our previous experience of TEAVR [2] endoscopic magnification, appropriate aortotomy, aortic margins traction, could provide in this case an excellent exposure to visualize the appropriate cutting of the base of the fibroelastoma. The patient presented an intra pericardial length of the ascending aorta superior to 6 cm and respected all the other selection criteria that we previously applied in TEAVR [5].

The encouraging intra and post-operative outcomes and fast recovery showed the potential benefits of a non-robotic totally endoscopic removal of PFE. We hope that this totally endoscopic surgical option will be reproducible in future similar cases, beginning from centres that already perform right minithoracotomy approaches for aortic valve surgery.

## Supplementary Information

**Additional file 1**. Video of the totally endoscopic aortic fibroelastoma resection.

## Data Availability

Data available on request from the authors.
